# Preparing an Insectary in Burkina Faso to Support Research in Genetic Technologies for Malaria Control

**DOI:** 10.1089/vbz.2021.0041

**Published:** 2022-01-13

**Authors:** Charles Guissou, M. Megan Quinlan, Roger Sanou, Robert K. Ouédraogo, Moussa Namountougou, Abdoulaye Diabaté

**Affiliations:** ^1^ Institut de Recherche en Sciences de la Santé-Direction Régionale de l'’Ouest (IRSS-DRO), Bobo-Dioulasso, Burkina Faso.; ^2^ Centre for Environmental Policy, Imperial College London, United Kingdom.

**Keywords:** biosafety, regulations, containment, mosquito, vector control, global health

## Abstract

The Institut de Recherche en Sciences de la Santé (IRSS) of Burkina Faso, West Africa, was the first African institution to import transgenic mosquitoes for research purposes. A shift from the culture of mosquito research to regulated biotechnology research and considerable management capacity is needed to set up and run the first insectary for transgenic insects in a country that applied and adapted the existing biosafety framework, first developed for genetically modified (GM) crops, to this new area of research. The additional demands arise from the separate regulatory framework for biotechnology, referencing the Cartagena Protocol on Biosafety, and the novelty of the research strain, making public understanding and acceptance early in the research pathway important. The IRSS team carried out extensive preparations following recommendations for containment of GM arthropods and invested efforts in local community engagement and training with scientific colleagues throughout the region. Record keeping beyond routine practice was established to maintain evidence related to regulatory requirements and risk assumptions. The National Biosafety Agency of Burkina Faso, Agence Nationale de Biosécurité (ANB), granted the permits for import of the self-limiting transgenic mosquito strain, which took place in November 2016, and for conducting studies in the IRSS facility in Bobo-Dioulasso. Compliance with permit terms and conditions of the permits and study protocols continued until the conclusion of studies, when the transgenic colonies were terminated. All this required close coordination between management and the insectary teams, as well as others. This article outlines the experiences of the IRSS to support others undertaking such studies. The IRSS is contributing to the ongoing development of genetic technologies for malaria control, as a partner of Target Malaria. The ultimate objective of the innovation is to reduce malaria transmission by using GM mosquitoes of the same species released to reduce the disease-vectoring native populations of *Anopheles gambiae* s.l.

## Introduction

Malaria remains a significant deterrent for economic development in affected countries, but more immediately, it is a primary cause of child mortality and health deterioration for adults (World Health Organization [WHO] 2020). Existing methods for reducing malaria transmission through vector control are limited and currently losing their efficacy owing to the increasing resistance of mosquitoes and parasites to insecticides and drugs, respectively (malERA Consultative Group on Vector Control 2011, Russell et al. 2013, Ranson and Lissenden 2016, Zhu et al. 2017).

The incidence of malaria has increased after several years of successful reduction in cases (WHO 2020). This has led to global recognition of an urgent need for additional methods in malaria control (WHO 2015, Alonso and Noor 2017). Genetic technologies hold great promise as an important addition to an integrated approach to reduction and, over time, elimination of malaria (Alphey et al. 2002, Matthews 2011, Gabrieli et al. 2014, Adelman 2015, Gantz et al. 2015). Target Malaria aims to develop and share innovative, cost-effective, and sustainable genetic technologies for malaria control.

In 2012, the Institut de Recherche en Sciences de la Santé (IRSS) became a formal partner of the research consortium later called Target Malaria. This followed several meetings with Imperial College London, which hosts the project, and consideration by both parties of a set of criteria such as identification of suitable field sites and experience managing complex projects to determine their suitability to work together (outlined in Quinlan 2019).

The genetic technology for malaria control under study by the IRSS and other Target Malaria partners is based on the eventual use of modified laboratory populations released to reduce the disease-vectoring native populations of *Anopheles gambiae* s.l. The phase of research reported here is to study a self-limiting transgenic mosquito strain, with the intention of eventually moving toward a persistent gene drive strain as the most cost efficient and effective final product from this research track (Burt 2014, Marshall and Akbari 2015).

In this regard, Target Malaria is following two types of stepwise development, or phased testing approaches, as recommended in various guidance. In addition to the stepwise approach of carrying out laboratory studies before moving to larger cage and controlled field studies (Coulibaly et al. 2015, WHO 2021), work has begun with a strain that has a sterile male trait and no gene drive (Windbichler et al. 2008), to best prepare the teams and test the systems for strains with different risk profiles such as with gene drive (Benedict et al. 2018, James et al. 2018). This stepwise approach also recognizes that experts in mosquito research likely have not faced the level of requirements for documentation and regulatory requirements that accompany a product development initiative, particularly one based on biotechnology.

Any open release of these genetically modified (GM) mosquitoes would occur only if the results in each step of the phased testing supports moving forward with that strain, after proper national regulatory review and permitting, and with adequate community acceptance. The attraction—and challenge—of using a genetic technology to reduce malaria transmission, however, is the sheer expanse of malaria endemic areas in Sub-Saharan Africa.

Sterile insect technique has proven effective in eradication or suppression of various arthropod pests, over large areas (Bellini et al. 2007, Vreysen et al. 2007, Dame et al. 2009, Dyck et al. 2021). A genetic technology using released laboratory-raised mosquitoes for area-wide control can provide benefits to inhabitants of a larger geographic area, in a more equitable social distribution (Brady et al. 2020, WHO 2020), while complementing many of the other malaria interventions (Coulibaly et al. 2015). This is important since malaria is most significantly a disease in rural areas and for the poorest communities (Barat et al. 2004) and access to individual or family-level solutions such as bed nets, repellents, appropriate housing, prophylactic drug treatments, or even reliable diagnosis and health services is limited for those most vulnerable.

Continual releases of sterile *Anopheles* mosquitoes, however, would entail huge production, release, and monitoring operations. Therefore, the innovation in genetic technologies is a mosquito construct that either reduces the vector population or prevents transmission of disease, which also persists and spreads without a continuous large area release: this is the impetus toward gene drive strains (Burt et al. 2018, Teem et al. 2020).

While these conditions point to a future including genetic technologies for reducing malaria transmission, the IRSS team knew that even greater preparation is needed when working with transgenic mosquitoes as a future control method than with other technologies not employing genetic modification, although area-wide mosquito control already requires multiple steps for preparation (Bartumeus et al. 2019, Ritchie et al. 2018, Oliva et al. 2021). This is due to the separate regulatory frameworks for biotechnology in most countries that became signatories to the Cartagena Protocol on Biosafety to the Convention on Biological Diversity, often referred to as the Cartagena Protocol (UNEP/CBD 2000), sometimes additional to other regulations, which were established with a focus on the precautionary principal (Pereira 2014).

The Cartagena Protocol provisions specifically exclude the use of the advanced informed agreement (AIA procedure) for imports for contained use (as discussed by Marshall 2010); however, Art18b specifies that any requirements for safe handling, storage, transport, and use are considered for shipments from laboratory to laboratory.

Although human health was not initially contemplated in that legal framework, interpretation has kept health interven-tions in the scope of the Protocol (Pereira 2014). Transgenic mosquitoes, for example, are an environmental intervention leading to a public health outcome. A recent African Union report highlights the various national entities involved in decision-making for innovative genetic technologies for malaria control, and the need for coordination among them (AUDA-NEPAD 2018). A higher level of preparation and management is needed as well because the novelty of the research indicates that earlier engagement is necessary in order for stakeholders to understand the technology and effectively contribute to consultations (Thizy et al. 2019).

Different cultural perceptions of the risks from use of modern biotechnology have led to a highly politicized debate on appropriate regulation internationally so that stakeholders internal to Burkina Faso may be exposed to considerable pressure from international opposition or advocacy groups, making direct knowledge even more important. Finally, the increase in discussion and wealth of publications considering gene drive mosquitoes (including the guidance for Arthropod Containment Guidelines in this scenario, presented in this volume) may confuse the situation reported here, in which a self-limiting transgenic strain with no gene drive or invasive traits is under discussion.

Therefore, the first import of transgenic mosquitoes to an African contained use facility was preceded by extensive preparations even before the IRSS submitted an application for permits. Preparations included not only the refurbishment of an insectary to ensure sufficient physical barriers for reliable containment but also the establishment of systems and documentation to demonstrate alignment with both project internal requirements based on international guidance, discussed below, and anticipated requirements based on a national legal review commissioned by Target Malaria.

All these activities required close collaboration between the country-level management team, the insectary team, the field entomology team, and others involved in stakeholder engagement, communications, legal compliance, finance, and so forth. Applications were submitted to the Burkina Faso biosafety authority, the Agence Nationale de Biosécurité (ANB), which evaluated the application for import into containment separately from the application for conducting studies there. The ANB then imposed specific terms and conditions necessary to achieve compliance with the permits. The IRSS, as a Target Malaria partner, complied with the terms and conditions of the permits and study protocols until the conclusion of studies, when the transgenic colonies were terminated. While this process is familiar to others working with GM insects, this article is to review experiences of completing this work as a first for the region and in a different context than where much of the international guidance has been prepared.

## Designing and Equipping the African Transgenic Research Insectary

When planning for a genetic technology, the transgenic insectary should be designed to provide sufficient production of mosquitoes to support field studies. This leads to a greater demand on the work systems than for smaller laboratory studies. Before moving to more natural conditions under a field study, the safety and, to the degree possible, potential efficacy of a candidate strain of transgenic mosquitoes will be determined within the laboratory setting (Mumford et al. 2018, see also James et al. 2020 for a discussion on safety and efficacy of gene drive mosquitoes). Comparisons between the laboratory colony and the target population of mosquitoes, such as for insecticide resistance, are best suited to take place inside a facility that is located in disease endemic countries.

**FIG. 1.**
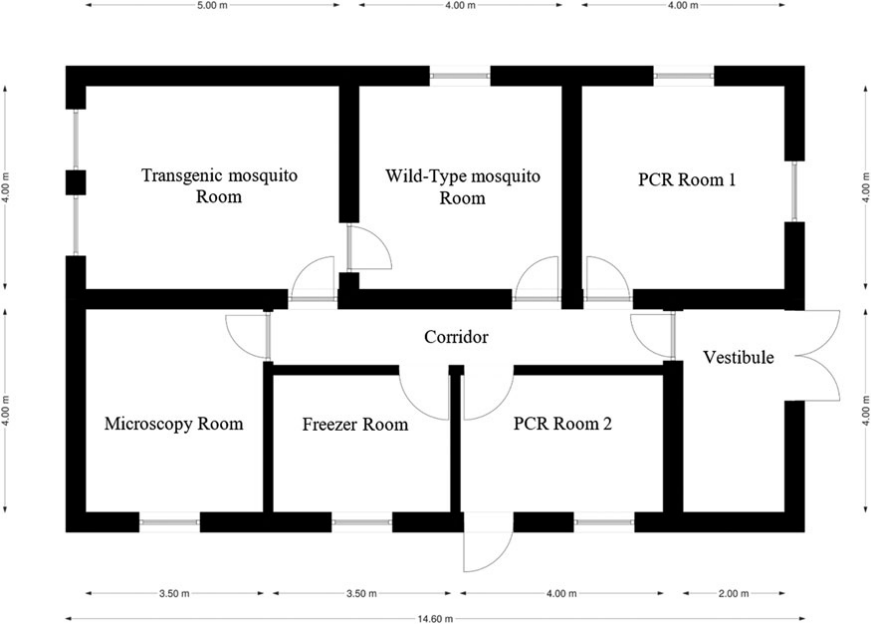
Floor plan of the IRSS facility. Location of insectaries along a corridor, with access through a vestibule with a double door entrance, clearly recognizable rooms for freezers, microscopes, PCR analyses rooms, each with its own entrance and an emergency exit. All windows in the facility are sealed or locked with restricted access to the keys. Note the spatial layout of the facility with an emphasis on containment measures (Target Malaria © All rights reserved). IRSS, Institut de Recherche en Sciences de la Santé.

In Burkina Faso, an unused building that required extensive refurbishment was selected for the future Arthropod Containment Level (ACL) 2 facility for housing and study of the transgenic mosquitoes (ACME and ASTMH 2019).* Figure 1 shows the floor plan of the IRSS transgenic insectary facility at the time of the import and associated studies.

The IRSS team worked with Target Malaria to define a process aimed at “facilities readiness”; this could be considered complying with or fulfilling all the various factors for biosafety as well as creating conditions for robust and reliable research before proceeding to an import permit application. The teams recognized that there are multiple requirements: maintaining biosafety and compliance with all other legal, institutional, funder, and partner policies; maintaining colonies of research mosquitoes that are fit and demonstrating consistent traits, to use in studies which provide reliable data for decision-making; and documenting evidence of these processes—especially given the aim of developing a strain of mosquitoes as a future product for malaria control (Quinlan et al. 2018a). A report on steps in preparing a design and construction or refurbishment of similar facilities appeared as Quinlan et al. (2018b).

Guidelines for working with mosquitoes should clearly indicate how to classify the work in terms of risk. For example, the level of management would relate to which mosquito species is under study and whether it is capable of vectoring disease that could be present in that setting. If the guidance is for mosquitoes that are not infected with the named pathogenic agents, care must be taken to ensure that this status is maintained (Table 1). Otherwise, the risk may become greater than the level managed through the design and operation of the insectary. Further preparations for facilities and procedures will be required as the research moves on to later stages of the phased development process (such as outlined in Adelman et al. 2017).

**Table 1.** Primary Strategies for Biosafety Employed in the Institut de Recherche en Sciences de la Santé Transgenic Insectary Facility

Objective	Example measures	Monitoring or confirmation
Preventing escape of mosquitoes	Physical barriers Purpose built design, stand-alone building Double door entry through vestibule Self-closing doors Windows sealed and resistant to breakage Ventilation ducts screened Treatment of waste Drains modified and filtered Solid waste autoclaved before being destroyed in external incinerator Control of free flying mosquitoes or other insects Light traps Weekly sweep checking under sinks, desks, etc. Stepped up monitoring if threshold passed Walls painted for easy detection Ant and other pest control Prevention of unauthorized removal Restriction of entry Limited number and required orientation for visitors	Routine inspection and occasional audits Scheduled maintenance and record keeping. Disposable filters not reused. No alternative waste streams Light traps. Recording of numbers and location for analysis of cause Biometric entry pad on door. Advance request for visitors, no one allowed without written approval. Register of those completing orientation. Register of those entering laboratory. Policies for no friends and family, and for lone workers
Preventing contamination of strains	Single project insectary, single transgenic strain held Species appropriate primary containers Harmonized labeling and handling system Numbers of male/female and transgenic/non are compared to expected rates Identify of imported strains confirmed routinely throughout generations and just before shipping Control of free flying mosquitoes (see above)	Funding policy, no exceptions Quality containers imported if necessary System if observed by colleagues and Manager and labeling is color coded and checked Database entries make any disparities easy to spot PCR testing and record keeping, shipping records cover topic on MTA
Preventing malaria in the insectary	Initial field-caught mosquito colonies follow SOP to segregate gravid females, combine only using larvae from eggs laid in containment and with PCR verification of family line identity (also prevents strain contamination) Blood for feeding kept clear of malaria Staff trained to identify and test quickly if symptoms arise from outside exposure Control of free flying mosquitoes (see above)	Laboratory notebooks with gels checked. A wild-type colony record requires each step to have been followed, to complete properly Use rabbit blood, drawn at the time needed by a qualified veterinarian who is monitoring the rabbits housed in a separate facility Test kits in place, medical staff available at work, policy to allow time off work

Adapted from Sanou et al. (2016).

MTA, material transfer agreement.

The Arthropod Containment Guidelines (ACME and ASTMH 2003) provide clear guidance for safe handling of arthropod vectors of human and animal diseases. Anecdotally, their thorough coverage and accessibility have made them a popular source for reference worldwide. These Guidelines, which were recently updated (ACME and ASTMH 2019), were developed as a reference for research laboratories to assess risk and establish protocols in relation to characteristics such as whether the species is endemic or exotic to the country or region of the research site.

When vector arthropods, in this case mosquitoes, are known to be free of the disease agent in question and are not exotic, an ACL 1 has been considered appropriate. However, if the strain or colony under study has been modified and is not yet approved for release, as in this case, ACL 2 is recommended to prevent exposure to the open environment (Scott 2005). The Guidelines have since been elaborated to give further guidance for their application in the case of transgenic insects with gene drive components (this volume). Table 1 includes some of the risk management strategies that the IRSS employs.

**FIG. 2.**
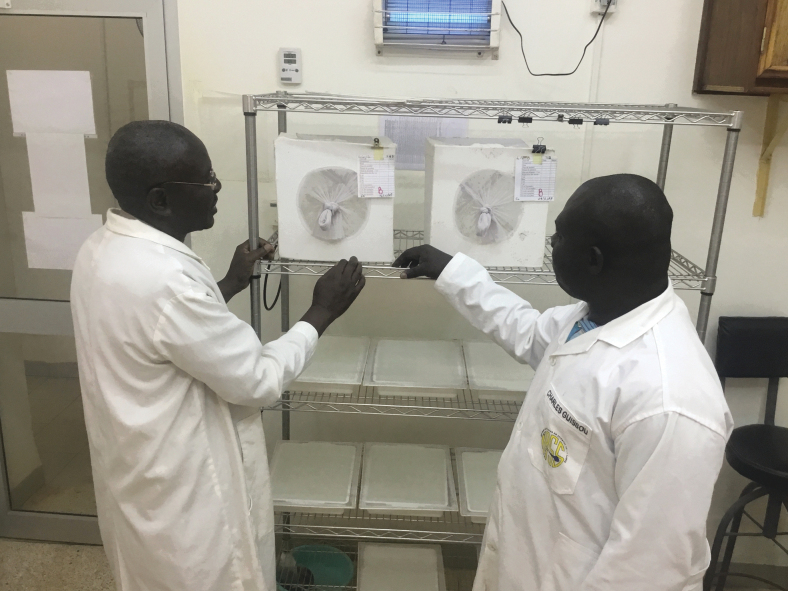
Checking containment measures of the ACL 2 insectary facility at the IRSS Bobo-Dioulasso in Burkina Faso. Adult mosquito cages with rearing and maintenance record cards attached. A UV light device is hanging on the wall whose function is to catch free flying mosquitoes outside the primary barrier of a cage. Mosquito larval trays for breeding mosquitoes are covered with two layers of net to avoid escapes (Target Malaria © All rights reserved). Pictured: (left to right) Sougrinooma Zoungrana (Senior Insectary Technician) and Charles Guissou (Senior Project Manager for Target Malaria Burkina Faso). UV, ultraviolet; ACL 2, Arthropod Containment Level 2. Color images are available online.

Many of the physical measures have complementary practices or require consumables. For example, the barriers to prevent loss of research organisms through sink drains, ventilation ducts, or doors must be coupled with waste treatment such as by autoclave. Regular checks and maintenance of such measures are required; filters must be changed on a routine basis. Figure 2 is inside the IRSS transgenic insectary, which comprises areas for mosquito rearing, studies such as insecticide resistance, microscopy for sorting the laboratory reared mosquitoes by sex and genetics, an area for DNA extraction and PCR, and other key components.

The design in Burkina Faso reflected a review of both international and national guidance for use of transgenic mosquitoes, not including a gene drive component. A transgenic containment laboratory depends not only on capital investments in infrastructure but also on appropriate institutional structure, trained staff (discussed further below), laboratory equipment, and supplies.

Guidance primarily from Europe, the United States, and Australia (*e.g.*, USDA et al. 2002, OGTR 2020 and Australian/New Zealand Standards 2010), is prepared in the context of institutions with long histories of managing biosafety and ethics. The type of institutional structures common to universities and research centers, such as Institutional Biosafety Committees (IBCs), in those settings cannot be assumed for a low- or middle-income country, although some individual institutions have prioritized resources for this. Therefore, the oversight and interface roles were replicated by Target Malaria until similar institutional arrangements were in place and/or verified (Kuzmina and Hoyle 2020).

To maximize local content of this research, equipment was purchased locally to the extent possible, but some was also imported during the refurbishment of the insectary. The typical measures to prevent damage from electricity surges and backup electricity generation in case of blackouts, critical for this research setting, were installed. The IRSS transgenic facility in Bobo-Dioulasso was fully renovated and ready for use in 2015 (Guissou et al. 2019). Key events in the preparation of the transgenic insect facility are presented in Table 2, alluding to the extensive commitment of resources and time for this phase of study.

**Table 2.** Key Events for the Target Malaria Burkina Faso Containment Facility Covering This Period

Key event	Year
Provide stored samples, protocol, and procedures for identification of the transgenic strain to the National Biosafety Laboratory in Burkina Faso	2020
Termination of the live transgenic colony and notification to the ANB	2019
Preparation and submission of final research report to the ANB	2019
Formal inspection of facility by the ANB	2019
Preparation and submission of interim research report to the ANB	2017
Research in the IRSS facility containment conducted with transgenic strain	2016–2019
Import of eggs of transgenic strain to contained use facility at the IRSS accompanied by the ANB inspectors	2016
Separate request to import transgenic strain submitted to the ANB and approval received	2016
Formal inspection of facility by the ANB	2016
Formal community acceptance for contained use	2016
Permission for contained use received from the ANB	2016
Submission of dossier to the ANB for contained use	2015
Import and training on non-GM color variant strain at the IRSS	2015–2016
Target Malaria audits	2015
Facility renovations to meet ACL 2 physical requirements and preparation of procedural biosafety and safety (fire, chemical, etc.) measures, SOPs and records for safe handling, and use of transgenic mosquitoes	2014
Establishment of *Anopheles coluzzii* colony from local field populations and colony record keeping	2014

ACL 2, Arthropod Containment Level 2; ANB, Agence Nationale de Biosécurité; GM, genetically modified; IRSS, Institut de Recherche en Sciences de la Santé; SOP, standard operating procedure.

## Establishing Procedures for Operating and Monitoring in the Transgenic Research Insectary

The IRSS insectary manager oversaw development of a Biosafety Manual, a regulatory requirement, which complements the standard operating procedures (SOPs) prepared for site-specific issues, including waste disposal, operation of the backup generator, and other methods for safe maintenance and operation of the contained insectary. There are also Target Malaria-wide SOPs for shipping, establishing a field caught colony, and handling the mosquito strains, to ensure that the mosquitoes are uninfected and provide consistency and common record keeping by all partners.

The IRSS team visited ACL 2 insectaries housing transgenic mosquitoes in other regions and reviewed SOPs from transgenic laboratories at Imperial College London. The final version SOPs and their implementation are reviewed in project team meetings, where experiences are shared. All the team is trained on any SOP before its application. The process of review is in line with Good Laboratory Practice (Adelman et al. 2017).

The Insectary Manager checks compliance using records linked with SOPs such as maintaining laboratory notebooks, following methods and equipment maintenance. This is complemented with a local and international team analysis of data from a purpose-made database covering fitness parameters, blood feeding, screening, import or export of strains, and so forth. Audits that are internal to the consortium but involve experts from outside the location so far have taken place when there is any change in the facility structure, management, a new mosquito strain with a different risk profile is to be imported, or other key events. This is in addition to official audits. These unofficial audits are based on extensive checklists and records of compliance, as well as observation of operations (similar to that described for field audits by Collins and Quinlan 2020).

Such methods for preparation are additional to any government's inspection and were developed for transgenic insectaries where there may not be institutional experience or possibly existing institutional committees covering these tasks. Even when these roles are well established, additional training and structured checklists may be welcomed to support work with new species or new forms of modification. Other approaches will be considered when moving to strains with different risk profiles, such as with gene drive components.

As part of operational preparations and regulatory requirements, the insectary team agreed on emergency procedures in case of any escape or conditions that could lead to escape of laboratory mosquitoes from the facility. Such a breach, however, would not occur unless there is failure of multiple barriers (including those shown in Table 1). A SOP describes a series of actions to rapidly destroy the colonies at each life stage, should the need arise.

The outdoors plan was based on baseline trapping of naturally occurring mosquitoes around the insectary over each season, which suggests low populations of *A. gambiae* s.l. in the immediate vicinity. Therefore, if an escape is discovered, the immediate response of the team is to quickly spray building exteriors and other potential resting sites within the immediate area surrounding the insectary, up to the perimeter walls of the campus near the insectary, with a registered insecticide. Others on campus, particularly those likely to be in the area (such as drivers waiting by vehicles), or researchers with other study organisms that could be affected if spray residue were carried into those laboratories were consulted about the plan.

Ongoing monitoring of various indicators for numbers and fitness of the mosquitoes is organized using a detailed database covering a range of indicators, primarily for the purpose of monitoring quality and performance (Mumford et al. 2018). This was developed for all of Target Malaria as a purpose-made data management and storage system. The data entered also provide a robust estimate of numbers of female adult mosquitoes in the laboratory colonies at the IRSS at any given time. This supports compliance over limits on the numbers in the facility and ensures relevance of assumptions in an independent risk assessment of the study strain (Hayes et al. 2018).

**FIG. 3.**
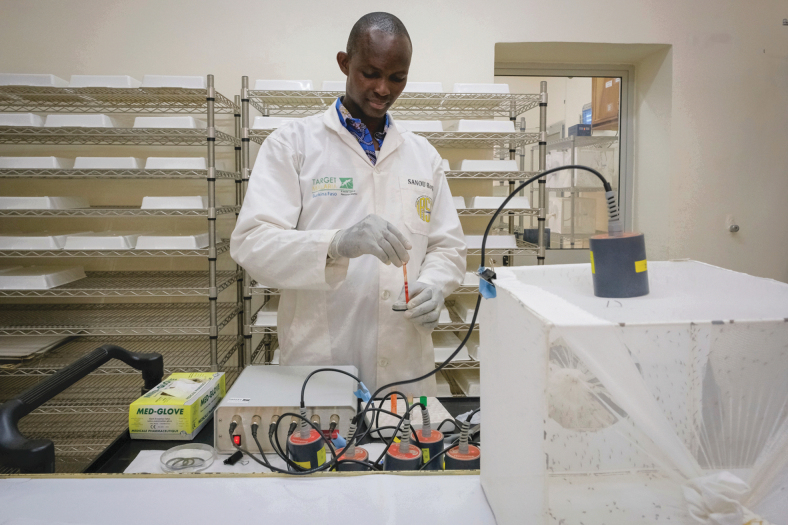
Setting up of the Hemotek^®^ blood feeding system of mosquitoes (transgenic and wild type) in the ACL 2 insectary facility at the IRSS, Bobo-Dioulasso in Burkina Faso. Filling a membranous chamber with rabbit blood using a pipette. Mosquitoes suck the blood through a Parafilm^®^ membrane. The cage contains female mosquitoes engorged on the membrane-fed blood meal (Target Malaria © All rights reserved). Pictured: Roger Sanou (Deputy Insectary Manager). Color images are available online.

There is also ongoing monitoring of temperatures and humidity within the rearing area to maintain a stable environment, at the desired levels for the research mosquitoes. The need for this and consistent diet for the larvae relate more to maintaining a colony quality than to biosafety, but the research will fail without these considerations. The Target Malaria policy is to rely on membrane feeding, as shown in Fig. 3, and this adjustment to local blood supplies, in this case from rabbits that are tested and managed by a trained veterinarian, also took some time. Such an adjustment period for the laboratory colony should be anticipated when setting the time period covered by any research permit. The field-caught wild strain also took some time to adjust to laboratory conditions. The imported transgenic strain needed multiple generations to build up numbers sufficient to proceed with studies.

The IRSS as a Target Malaria partner carried out extensive engagement with the community surrounding the campus where the insectary is located, in addition to stakeholder engagement at the selected field sites according to the recommendations of the WHO (2021) and reported by Pare Toe et al. (2021).

## Ensuring Capacity of the Research Team

The insectary team was created with highly experienced mosquito experts and laboratory technicians. As the preparation of the transgenic insectary facility was iterative, similarly to other West African experiences of fully achieving the level needed for clinical trials (Guindo et al. 2012), the composition and roles of those in the team have been iterative. The demands of coordination among the various teams relying on the insectary, with the regulators, the funders, and so forth, led to additional staffing for management roles.

Maintaining the laboratory colonies requires rigorous attention, in particular because of the need to backcross each generation due to male sterility of the strain. The maintenance of a naturally occurring (nontransgenic) color variant strain before this import (Sylla et al. 2017, Quinlan et al. 2018b) was another way to test the system and to increase capacity of the teams. As the renovation of the facility progressed, an on-site audit was conducted by a team of experts contracted from within the project to review compliance with containment standards and subsequent visits to review operations took place in advance of applying for an import permit (Quinlan et al. 2018b). This facilitated further improvements and built confidence within the insectary team in anticipation of an official inspection of the insectary by the ANB before approving the study permit.

In addition to the internal training noted above, meetings with the ANB officers, with personnel from the New Partnership for Africa's Development (NEPAD) of the African Union's Development Agency and other regulatory experts, have given the IRSS team insights into regulatory requirements and the process in Burkina Faso in particular. This learning process included discussions on how to ensure compliance and maintenance of biosafety measures in the insectary. A routine meeting of all the IRSS teams continues as part of the culture and support of biosafety compliance. There is an agenda item for training updates and on insectary operations and biosafety measures, so that the topic is always in the forefront for the entire country team.

Stakeholder engagement with the community around the insectary was conducted by a specialist team but draws on the expertise and time of the Insectary Manager and Senior Project Manager in particular. This included a period of knowledge sharing between the project and stakeholders, with visits to the transgenic insectary (Swetlitz 2017). A video of the facility, prepared as part of the community outreach, is available on the Target Malaria website and shows some of the biosafety measures, as well as several routine operations for maintaining a colony and sorting larvae (Target Malaria 2018). A close relationship with colleagues who offer other types of expertise is essential to achieving a successful program (Thizy et al. 2019).

## The Importance of a Functioning Biosafety System

The Cartagena Protocol established a widely endorsed framework for decision-making around transboundary movement of living modified organisms (see Pereira 2014 and his upcoming revision for discussion). National systems for risk-based decisions on transgenic organisms therefore arose from that harmonized approach to biotechnology but vary by the underlying understanding and interpretations that arise and evolve at the national or regional level (Romeis et al. 2020), and the institutional structure often differs.

The African Biosafety Network of Expertise (ABNE) of the NEPAD assisted in setting up a functional biosafety system in a number of African countries (ABNE 2016) and continues to interface with the regulators, scientists, and stakeholders for the regulatory process related to transgenic mosquitoes. The ANB became a leader in biosafety regulation in the region, drawing on their experience from *Bt* (*Bacillus thuringiensis*) cotton (Vitale et al. 2010, World Bank 2015). The IRSS thereafter benefited from operating within an already established and tested regulatory system, although one not developed with GM insects or public health objectives as the focus.

The ANB reviewed the plans for the insectary, and visited the site after its preparation, as part of the evaluation (described in Decret No 2015-215, Government of Burkina Faso 2015). An important part of maintaining restricted access to a containment facility is to require advance permission to be sought for entry and to complete an orientation with all visitors in advance of entry, and the IRSS followed the same process for the inspection as for other visits, to demonstrate the preparations for restricted access and what other visitors would experience.

A permit (ANB 2016a) was granted on April 12, 2016, allowing studies to take place on a transgenic sterile male strain of mosquitoes; the permission was granted exclusively to the IRSS laboratory discussed in this article, and not to the overall institution or other sites. The ANB then made a recommendation concerning import of the research strain, and the Ministry of Higher Education, Scientific Research and Innovation followed on October 21, 2016, with approval for import into containment, exclusively to the IRSS laboratory discussed in this article and for a specific protocol described in the application, for a specific time period (ANB 2016b).

Since the time the permits were granted, the IRSS complied with the terms and conditions including to inform the ANB of any changes in conditions, until the completion of the research when the colonies from the transgenic strain were terminated. Burkina Faso employs a system of monitoring compliance that relies on the institutional officers and insectary facility management to conduct and document ongoing checks for periodic review by the government, much like the systems in Italy and France (Quinlan 2019).

## The Import of Transgenic Mosquitoes to the IRSS

**FIG. 4.**
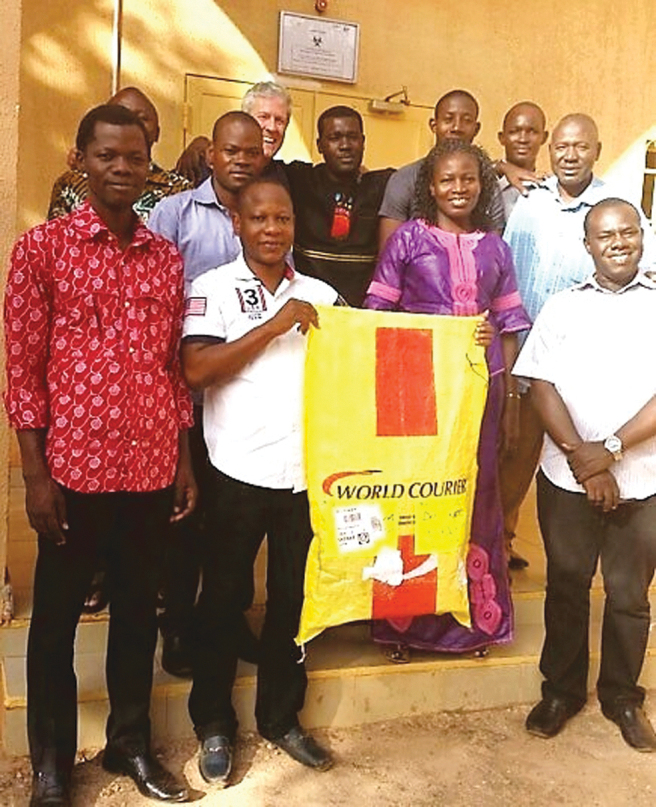
The IRSS transgenic insectary team receives the first import of transgenic mosquitoes to Africa for research in malaria control (Target Malaria © All rights reserved). Pictured: (left to right) *top row*: Wilfrid Meda, Mark Q. Benedict (CDC Foundation), Abdoulaye Diabate (Principal Investigator for Burkina Faso), Fulbert Zoungrana, Roger Sanou, Sougri-Nooma Zougrana; *bottom row*: Robert K. Ouédraogo, Anselme Ky, Moussa Namountougou, Lea Pare/Toe, Charles Guissou. Color images are available online.

The first import of transgenic, non-gene drive mosquitoes arrived at the IRSS insectary on November 3, 2016. A photograph documenting this significant step is presented in Fig. 4.

Shipping of live insects, even in a vulnerable life stage such as mosquito eggs, requires particular packaging, handling, and labeling to meet international guidance (IATA 2020). To test the shipping system that maintains a chain of command and must deliver the live mosquito material before it loses quality or perishes, Target Malaria carried out several trial shipments using nontransgenic mosquito material as part of preparations for import of the regulated mosquito strain, which allowed the courier companies to gain experience with this category of shipment. While it is important to establish a relationship with professional courier services, having more than one company providing the service, especially if they use different routes, is one way to manage the possibility of no service at any given time.

The strain studied in this phase was developed using a G3 laboratory strain background, a widely used research resource. The transgenic strain resulted from genetic modification by researchers at Imperial College London to produce dominant sterile male offspring. This strain was then evaluated and maintained by the Target Malaria team at the University of Perugia, which since has transferred to the Polo GGB facility in Terni, Italy.

The transgenic strain was imported to Burkina Faso from Italy with the necessary shipping forms, including the authorization from the ANB, and a Target Malaria internal certificate declaring the genetic identity of the strain and how it was confirmed, as part of the Material Transfer Agreement. Upon arrival, the strain was backcrossed in the transgenic insectary facility to a wild-type strain established from field-caught local *Anopheles* populations, managed to be free of malaria infection (as outlined in Table 1), and maintained in the laboratory for several generations.

## Discussion of the Experience

The IRSS insectary team benefited from a high commitment at both local and Target Malaria level management toward biosafety and capacity building. Significant time and resources were allocated over the course of this phase of the research to ensure that training, proper laboratory procedures, additional documentation, and data collection took place both before the import of the transgenic strain and after import and introgression of the genetic modification into the existing, field-caught laboratory colony.

The overall institution responded to the need for an IBC, which should benefit future research on biotechnology at the IRSS. Biosafety professionals in other settings have been challenged by the number and complexities of cases employing containment studies of GM insects (O'Brochta et al. 2020). The role of IBCs remains central to the process of scientific debate and oversight (Heitman et al. 2016), despite these challenges. The transgenic insectary studies at the IRSS were an early step in the innovation of genetic technologies for malaria control. At the same time, the entire outcome in Burkina Faso relies on the IRSS insectary team and the mosquitoes produced there for both laboratory and field studies.

The researchers also benefited from being under an experienced regulatory system. Indeed, Burkina Faso has become a source of expertise for other countries building their functional biosafety systems. The biosafety decision-making system in Burkina Faso had been tested previously by the *Bt* cotton experience (Vitale et al. 2010, Dowd-Uribe, Schnurr 2016). While aligned with international intergovernmental treaties and protocols, the Burkina Faso system is based on the country's own understanding of the potential benefits and risks from modern biotechnology. The advance discussions allowed the IRSS to learn how to comply with the requirements of the biosafety system. The experience reported here has primed African regulators to look forward to future technologies and contribute early to the global discussions on potential use of gene drive (Burt et al. 2018).

This entire process of preparing and managing a transgenic mosquito insectary was very intensive and required additional management staffing than originally envisioned. The import and studies with the sterile male transgenic strain was an important step for the IRSS and Target Malaria in capacity building and for understanding and using the regulatory and stakeholder engagement systems. This established a stepwise process that included study organisms with no persistence in the environmental before persisting or invasive ones, as well as the steps from laboratory studies to field studies.

After safety and efficacy studies in the containment facility were completed in this first phase of research on the self-limiting sterile male strain, the IRSS sought permission from the ANB to carry out a controlled field release of this same strain (Pare Toe et al. 2021, Target Malaria 2019). The initial preparations for the import and laboratory studies of this transgenic strain provided the foundation for this further step, and the same insectary provided the mosquitoes used in the field release.

Much of the guidance on transgenic insectary facilities is from sources external to disease endemic countries and has been adapted to African settings. The West Africa Integrated Vector Management Steering Committee is drafting a series of guidelines relating to transgenic mosquitoes but also potentially what is needed for working with gene drive mosquitoes (AUDA-NEPAD 2020). This reportedly will include guidance on stakeholder engagement, IBCs and containment laboratories, as well as other topics in anticipation of future use of gene drive-based vector control technologies. Our article should fill the gap for those working with transgenic mosquito without a gene drive or invasive component.

## Conclusions

The IRSS experience is from the perspective of a government-funded research institution working toward a potential innovation in the fight against malaria. This public health objective, and the fact that the technology is held by a not-for-profit consortium with commitments to global access in the future, could affect public acceptance of the technology. However, this does not alter the regulatory requirements and related scrutiny that comes with managing a transgenic insectary facility working toward an innovation for malaria control. The scrutiny is even greater due to the research group's stated plans to work with gene drive in the future, if permitted.

This article describes initial work toward establishing a containment facility for import and study of transgenic mosquitoes, without any gene drive traits. This experience has solidified the IRSS team capacity—including management capacity—for working with transgenic mosquitoes and should be recognized as a crucial step in preparing for research on other mosquito strains that have greater potential for widespread vector control. The authors hope that others can learn from and build on the Burkina Faso experience, which complements guidelines and advice from outside Africa.

## Notes

^*^
The experiences reported here reflect work on a transgenic mosquito line that does not include any gene drive components. While future use of gene drive mosquitoes is mentioned, the described process and measures only relate to GM mosquitoes not including gene drive, unless otherwise stated.
